# Radiation Therapy Combined With Checkpoint Blockade Immunotherapy for Metastatic Undifferentiated Pleomorphic Sarcoma of the Maxillary Sinus With a Complete Response

**DOI:** 10.3389/fonc.2018.00435

**Published:** 2018-10-17

**Authors:** Kripa Guram, Maria Nunez, John Einck, Loren K. Mell, Ezra Cohen, P. Dominick Sanders, Sayuri Miyauchi, Elizabeth Weihe, Razelle Kurzrock, Sarah Boles, Andrew B. Sharabi

**Affiliations:** ^1^Department of Radiation Medicine and Applied Science, UC San Diego Moores Cancer Center, San Diego, CA, United States; ^2^Division of Experimental Therapeutics, UC San Diego Moores Cancer Center, San Diego, CA, United States; ^3^Division of Hematology and Oncology, Center for Personalized Cancer Therapy, UC San Diego Moores Cancer Center, San Diego, CA, United States; ^4^Department of Radiology, University of California, San Diego, San Diego, CA, United States

**Keywords:** head and neck cancer, sarcoma, radiation, stereotactic radiotherapy, immunotherapy, checkpoint blockade

## Abstract

**Background:** Undifferentiated pleomorphic sarcoma (UPS) of the maxillary sinus is an extremely rare malignancy of the head and neck. Surgery is the mainstay of treatment for UPS; however, proximity to vital structures makes it challenging to achieve negative surgical margins. Adjuvant therapy including radiation therapy with or without chemotherapy is generally indicated. Despite advances in multimodality treatment, objective response rates to available therapies and prognosis of metastatic UPS remain dismal. Immunotherapy has become a fourth cornerstone of cancer therapy and checkpoint blockade immunotherapy is a standard of care for recurrent or metastatic cisplatin-refractory head and neck squamous cell carcinoma. Checkpoint blockade immunotherapy is being studied in metastatic sarcoma, including UPS, and while initial results are promising, objective response rates remain below 20%. However, adding radiation therapy to checkpoint blockade immunotherapy has been shown, in both preclinical and retrospective clinical studies, to have combinatorial effects on both local and metastatic disease. Thus, further investigation into the effects of radiation therapy combined with immunotherapy in head and neck sarcomas is warranted.

**Case Presentation:** We present a case of metastatic, chemotherapy-refractory, UPS of the maxillary sinus in a 55-year-old male treated with checkpoint blockade immunotherapy combined with radiation, which resulted in a complete response.

**Conclusions:** This is the first report to our knowledge of metastatic UPS treated with a combination of radiation and dual agent checkpoint blockade immunotherapy. Further investigation is warranted to study the effects of this combination in patients with metastatic UPS that fail to respond to currently available therapies.

## Background

Sarcomas are rare and diverse malignant tumors originating from mesenchymal tissue, accounting for < 1% of all adult malignancies (1, 2). Soft tissue sarcomas (STS) that lack a line of differentiation or characteristic immunohistochemical, genetic, or histologic features of existing categories of sarcomas now fall under undifferentiated/unclassified sarcoma, a new category introduced in the 2013 WHO classification (3). Undifferentiated pleomorphic sarcoma (UPS), a subset of undifferentiated/ unclassified sarcoma, includes high grade pleomorphic malignant tumors and is essentially a diagnosis of exclusion. Each year in the United States, there are fewer than 300 new cases of adult head and neck UPS (4).

Surgical resection is the mainstay of therapy for head and neck UPS, but local recurrence (LR) rates exceed 31%. Adjuvant chemotherapy and radiation can decrease LR (5, 6), but metastatic spread can occur and treatment options are limited.

While the incorporation of immunotherapy into the management of sarcomas is lagging behind other tumor types, STS was the first disease reported to have been successfully treated with a form of immunotherapy in 1891 by Coley (7). Modern immunotherapy is now being studied for metastatic STS, and early results are encouraging (8). Checkpoint blockade immunotherapy (CBI) works by inhibiting the negative regulators of immune activation (immune checkpoints) that restrict anti-tumor responses, including the cytotoxic T lymphocyte-associated protein 4 (CTLA-4) and programmed cell death (PD-1/PD-L1) pathways. Unfortunately, durable complete responses with CBI are rare (9).

## Case presentation

A 51-year-old Caucasian man presented to his primary care provider with left “facial pressure” and pain radiating to his nose. After two failed courses of antibiotics and steroids, CT scan 6 months after initial presentation revealed a left maxillary mass, and biopsy showed undifferentiated pleomorphic sarcoma (UPS) (stage cT2bN0M0, FNCLCC grade 3/3). PET/CT demonstrated a 5.1 × 4.7 cm infiltrative mass centered at the left maxillary sinus and nasal cavity with bony destruction extending into the left orbit, ethmoid sinuses, and inferotemporal fossa and no evidence of metastatic disease.

Neoadjuvant chemoradiation with doxorubicin and 30Gy radiation therapy (RT) in 10 fractions to the left maxillary sinus (modified Eilber regimen) was followed by radical resection 9 months after initial presentation (10). Pathology showed a 3 cm tumor with multiple positive margins, which prompted the addition of post-operative boost RT to 26Gy in 13 fractions to the tumor bed. Follow-up PET/CT immediately after boost RT showed fluorodeoxyglucose (FDG) avid lesions in both the tumor bed (SUVmax = 5.1) and left submandibular neck (SUVmax = 32.1). Subsequent ultrasound-guided biopsy of a left submandibular neck mass was positive for high-grade sarcoma.

After four cycles of gemcitabine and docetaxel, five of five lymph nodes were negative for disease on surgical lymph node dissection of the neck. After completing two additional cycles of chemotherapy, surveillance imaging showed no evidence of disease for 23 months off of treatment until CT scan detected multiple new, large lymph nodes in the left level V area of the neck and supraclavicular region as well as a left apical extrapleural mass. Biopsy of the left neck mass showed UPS. Foundation One CDx (Foundation Medicine, Cambridge, MA) immunohistochemistry profiling showed low-positive PD-1 and PD-L1 expression (1+ staining intensity, 1–24% staining distribution) on tumor infiltrating lymphocytes (TILs), and high-positive PD-L1 expression (2+ staining intensity, >25% staining distribution) on tumor cells (Supplemental Figure A).

**Figure 1:**
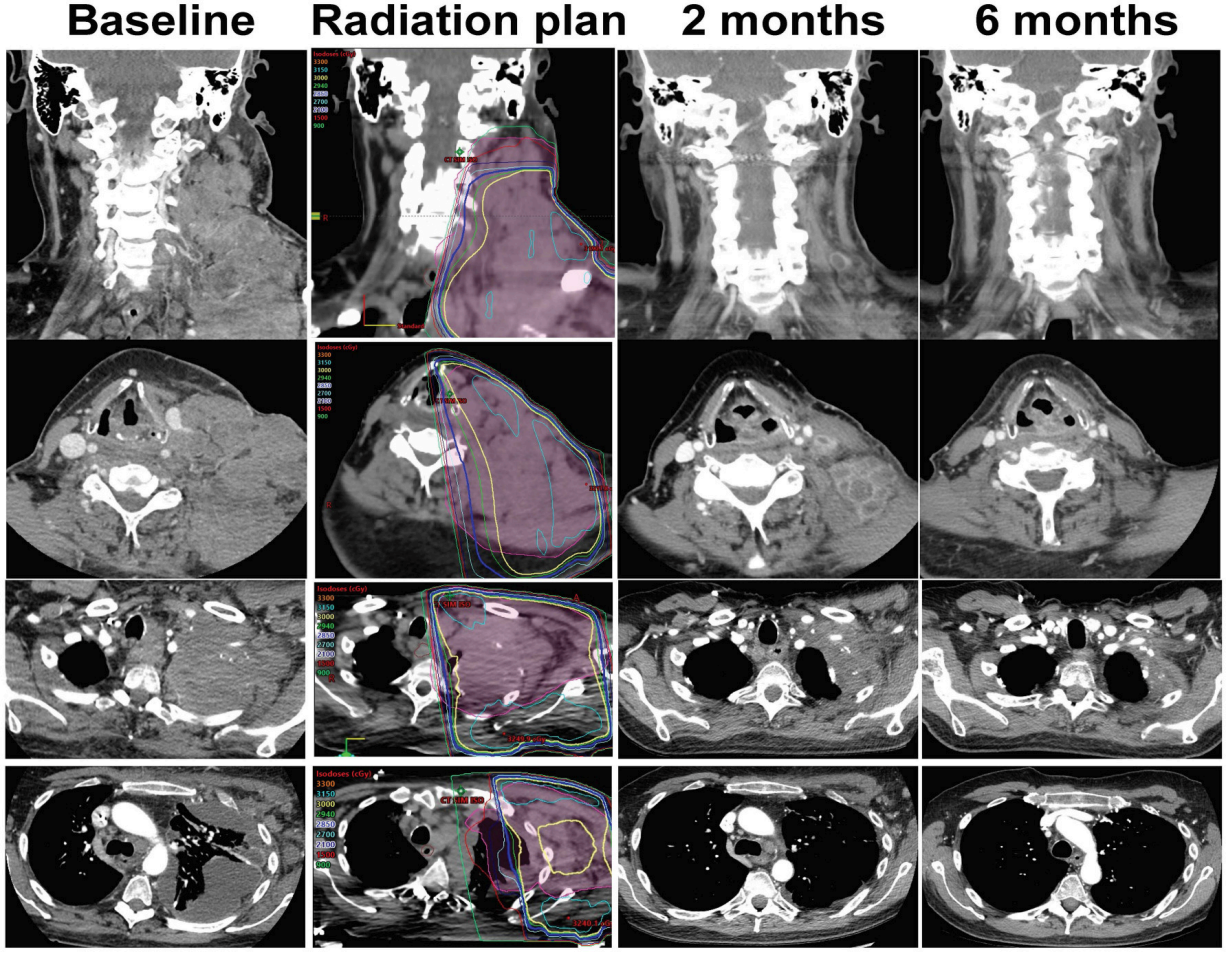
CT imaging of patient treated with RT plus nivolumab. Excellent partial response at 2 months and near complete response at 6 months of the lesions in the left neck, supraclavicular region, lung apex, pleural, and axillar disease. Response was maintained for 13 months.

His disease continued to progress on PET/CT despite two additional cycles of gemcitabine plus docetaxel, as well as three cycles of ifosfamide (Figure 1). Significant symptomatic progression of disease in the neck prompted treatment with palliative radiation therapy combined with biweekly intravenous nivolumab. The first dose of nivolumab was given 11 days prior to 30Gy RT to the left neck mass delivered in 10 fractions. Follow-up CTs demonstrated a striking decrease in tumor burden at the left lateral neck mass, supraclavicular lymph nodes, left chest wall and left axilla over the next 13 months of nivolumab treatment (Figure 1). Additionally, serum lactate dehydrogenase (LDH), a prognostic factor and surrogate marker of disease burden, decreased from 1,000 U/L at the beginning of this therapy to 215 U/L (11) (Figure 3).

CT scan of the neck 13 months after initiating palliative RT plus nivolumab showed an increase in the size of a right tracheoesophageal groove lymph node at the margin of the radiation field and redemonstration of an enlarged node left clavicular node. The patient was enrolled on a phase II randomized clinical trial combining stereotactic body radiation therapy (SBRT) with CBI (NCT02843165), and randomized to the SBRT plus CBI treatment arm. He was treated with dual agent CBI including ipilimumab and nivolumab, receiving 24Gy SBRT in 3 fractions to the right tracheoesophageal groove lymph node 1 week after the first of four intravenous ipilimumab infusions (1 mg/kg), followed by three more ipilimumab treatments Q3 weeks.

**Figure 2:**
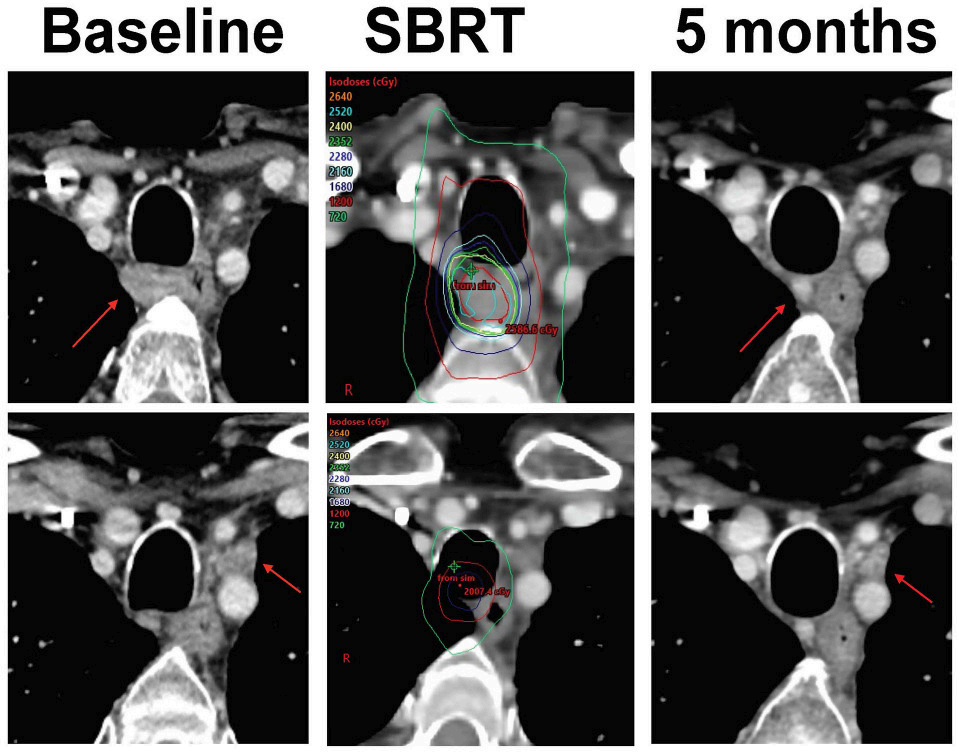
CT imaging of patient with Stereotactic Body Radiation therapy (SBRT) plus ipilimumab and nivolumab. Resolution of right sided tracheoesophageal lymph node and enlarged node between the left common carotid and subclavian arteries. The patient had no new lesions and no measurable disease by RECIST criterion consistent with a complete response. Patient has remained in complete response with continued decrease in size of these lymph nodes as of most recent imaging 2 years after his initial treatment with palliative RT combined with nivolumab.

Five months after starting SBRT plus dual agent CBI, CT imaging of the neck showed resolution of the irradiated right tracheoesophageal groove lymph node (Figure 2) as well as the non-irradiated left clavicular node enlargement and no new or progressive lymphadenopathy or other masses. He had previously developed hypothyroidism on nivolumab alone, but treatment with SBRT plus dual agent CBI was well-tolerated without any high-grade toxicities. Assessment by Response Evaluation Criteria in Solid Tumors (RECIST) 1.1 5 months after initiation of ipilimumab plus nivolumab plus SBRT determined complete response. The patient remains in complete response at most recent imaging over 2 years since his initial treatment with radiation combined with nivolumab and he continues to do well clinically (Figure 4).

**Figure 3:**
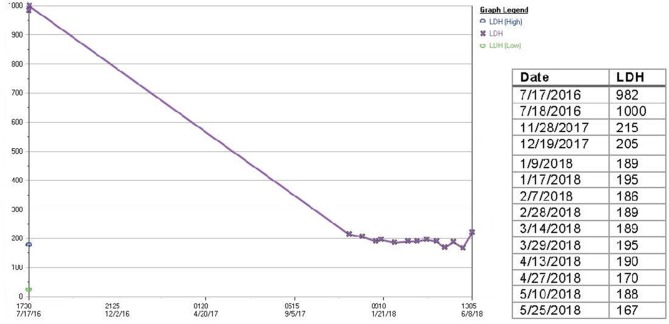
Serum LDH of patient treated with RT plus nivolumab. Serum LDH decreased from 1,000 U/L at the beginning of therapy to 215 U/L at 4 months. Serum LDH has been maintained within normal limits (125–250 U/L) until present.

**Figure 4:**
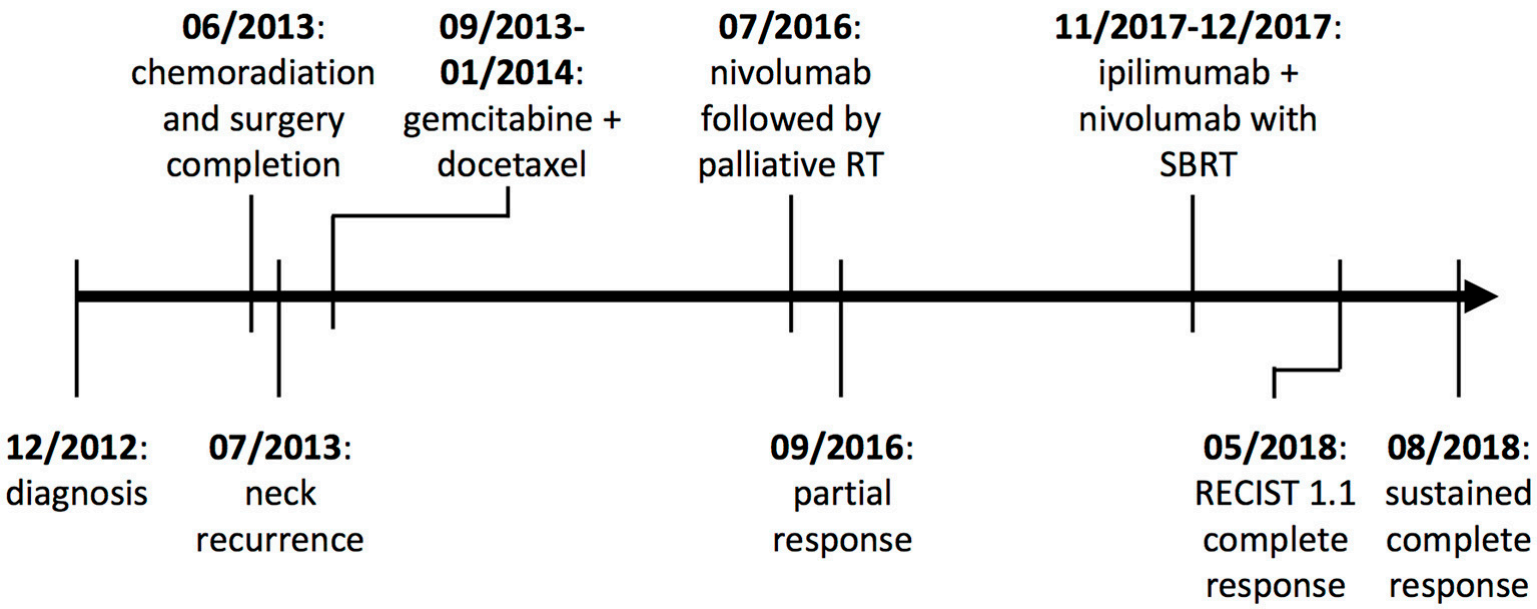
Treatment timeline. Treatments and responses from diagnosis until present.

## Discussion

Metastatic head and neck UPS is a very rare disease associated with a median survival of 14–17 months (12). Chemotherapy is the mainstay of treatment for metastatic disease and enrollment in a clinical trial is preferred when available.

CBI is an experimental and potential future treatment option for advanced UPS. In a recent study, Pollack et al found greater expression of genes related to antigen presentation and T-cell infiltration in UPS compared to other types of STS (13). These findings are consistent with the outcomes observed in the phase II SARC028 trial studying pembrolizumab in patients with sarcoma, including 10 patients with UPS. SARC028 showed an overall objective response rate (ORR) of 18% and 12-week PFS of 55% with pembrolizumab, with 40% ORR in UPS (*n* = 10) (14).

Nevertheless, the majority of patients do not have an objective response to single agent CBI. Current strategies to enhance response rates and durability include using dual agent CBI and combining CBI with RT. In a multicenter phase II randomized trial, 85 patients with advanced sarcoma who failed prior therapies were treated with nivolumab ± ipilimumab. Only two of 38 patients, including 0 of 5 with UPS, had confirmed responses to nivolumab alone (median PFS 2.6 months), compared to six of 38, including 2 of 6 with UPS, in the combined therapy group (median PFS 4.5 months) (15). The study concluded that nivolumab alone has limited efficacy in unselected sarcoma populations.

Data also suggest that combination therapy with RT plus CBI can improve disease control and progression-free survival (16, 17). The systemic regression of metastatic lesions after local irradiation of a single lesion, known as the abscopal effect, was first observed many decades ago and has been shown to be mediated by the immune system (18). RT increases anti-tumor immunity by upregulating antigen and costimulatory signal expression on tumor cells, shifting the cytokine profiles, and recruiting immune effector cells and antigen-presenting cells to the tumor (19).

Keung and colleagues found that patients' UPS tumors treated with neoadjuvant RT had increased tumor associated CD4+ T cells, and CD8+ T cells (*n* = 17). Furthermore, 21% of tumors stained positive for PD-L1 after treatment with RT, compared to 0% at baseline (20). These data suggest that RT could alter the tumor microenvironment and potentially enhance the activity of CBI in UPS. PD-L1 expression on both tumor cells and TILs has been associated with higher probability of response to checkpoint blockade immunotherapy in multiple tumor types, but this has yet to be shown in sarcoma and further studies are needed to identify reliable predictors of response (21). Ongoing clinical trials are studying combined RT plus CBI for UPS (NCT03116529, NCT03307616, NCT03092323).

## Conclusion

Metastatic UPS of the head and neck is an aggressive disease with poor prognosis. An ORR of <20% in any type of metastatic STS treated with any approved therapy highlights the need for additional options. We report a rare case of metastatic UPS originating from the maxillary sinus that progressed on multiple systemic therapies. The patient achieved a complete response on combination RT plus dual agent CBI. To our knowledge, this is the first report of radiation combined with dual agent CBI in UPS. Ongoing randomized studies will elucidate the benefit of radiation therapy combined with CBI.

## Ethics statement

Ethics approval and consent was obtained for the preparation and publication of this study through the UCSD Human Resource Protection Program via IRB approved study HRPP 151571. Consent for publication: written consent for publication was obtained from the patient discussed in this case using an institutional consent form.

## Author contributions

KG: acquired data, wrote, and revised critically for important intellectual content; MN: acquired data, revised critically for important intellectual content; JE, LM, and SB: treating physician, aquired data, revised critically for important intellectual content; EC and RK: substantial contributions to the conception or design of the work; revised critically for important intellectual content; PS, SM, and EW: acquired data, revised critically for important intellectual content; AS: substantial contributions to the conception or design of the work, treating physician, principle investigator of clinical trial, acquired data, revised critically for important intellectual content.

### Conflict of interest statement

EC reports research funding from Pfizer, Merck, AstraZeneca, and Bristol-Myers Squibb outside the submitted work. RK reports personal fees from X-Biotech, personal fees from Actuate, other from Genentech, other from Pfizer, other from Sequenom, other from Guardant, other from Foundation Medicine, other from Merck Serono, other from CureMatch, outside the submitted work. AS reports research funding from Varian Medican Systems and Pfizer. The remaining authors declare that the research was conducted in the absence of any commercial or financial relationships that could be construed as a potential conflict of interest.
