# Supplementary figures and images for: Radiation Therapy Combined With Checkpoint Blockade Immunotherapy for Metastatic Undifferentiated Pleomorphic Sarcoma of the Maxillary Sinus With a Complete Response
Source: Front Oncol. 2018 Oct 17;8:435. doi: 10.3389/fonc.2018.00435 (PMC6199376; doi:10.3389/fonc.2018.00435)

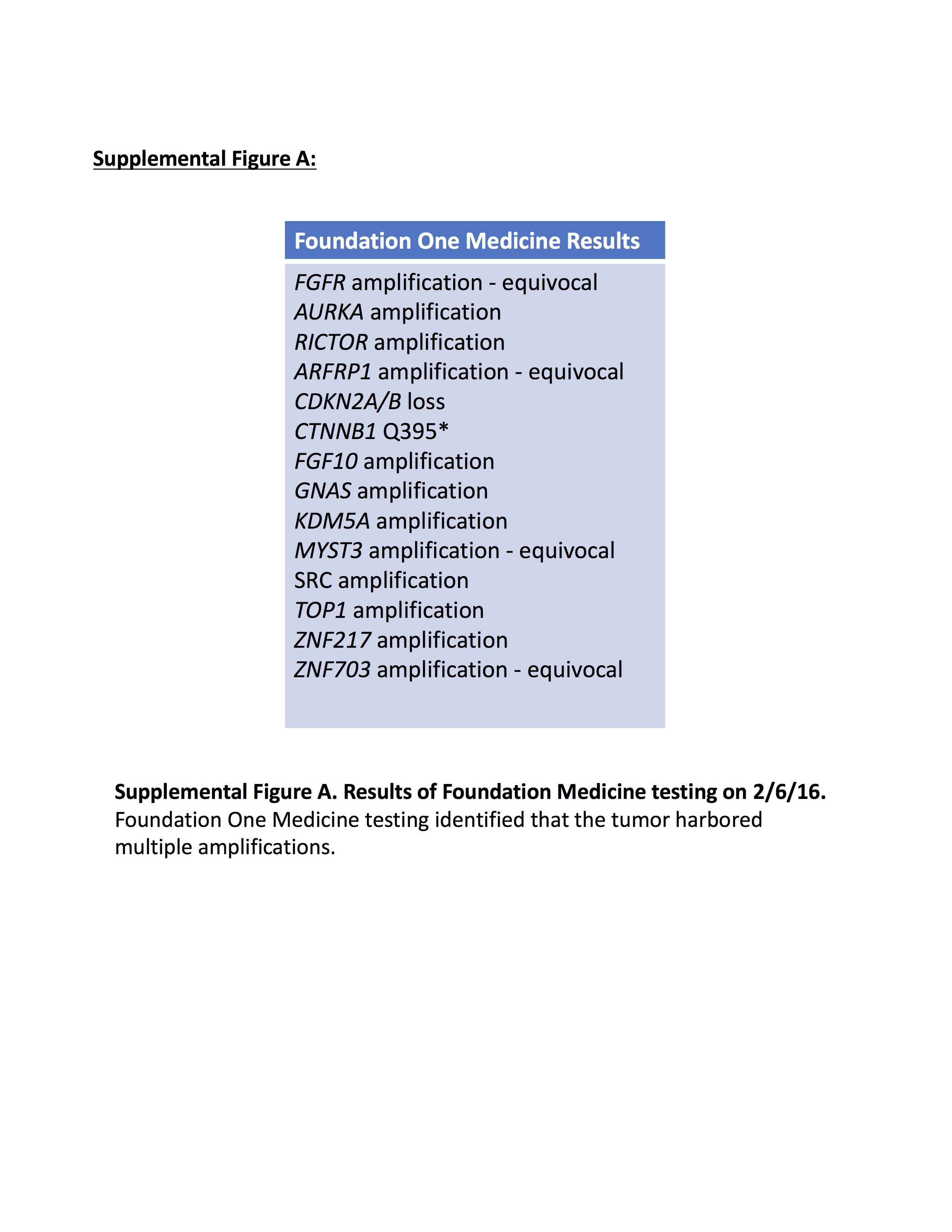

Supplement: Supplementary file 1 [file Image_1.JPEG]
